# Supplementary material for: ‘We are not the virus’–Experiences of racism among East & Southeast Asian heritage young people in London during the height of the COVID-19 pandemic
Source: PLOS Glob Public Health. 2024 Jan 24;4(1):e0002016. doi: 10.1371/journal.pgph.0002016 (PMC10807763; doi:10.1371/journal.pgph.0002016)
Supplement: S1 Text — (DOC) [file pgph.0002016.s001.doc]

## Interview schedule - The experiences and impact of COVID on ESEA heritage young Londoners

## Aims and Objectives:

## Investigate ESEA young Londoners’ experiences of the pandemic (particularly COVID-related racism), and the impact on their families, education, health, and social identities;

## Explore ESEA youth perspectives on the pandemic, relating to the role of education and health in their everyday environment and society;

## Relate findings to broader issues of race, inequalities, education and health relating to the impact of COVID-19 in London and beyond.

## Family and self

1. Can you tell me your name and how old you are?
2. Could you tell me which part of London you live in? How long have you lived there?
3. Could you tell me who lives with you at home? (parents/guardians, siblings, grandparents, etc.; age of other people)
4. Could you tell me where you were born?
5. Could you tell me where your parents were born?
   (*If parents born in the UK*: Where your grandparents were from? When they moved to the UK?)

## School & learning experiences

1. What school do you go to? (name/area/type of school, how far from home, etc.) What year are you at school?
   (*If they are over 18/finished school by last summer, ask about their experience last year or current education situation)*
2. Could you describe to me what kind of pupils are at your school? Are there any other pupils who are also ESEA at your school? (probe: ethnic backgrounds and class)
3. Do you see yourself as a good, average, not so good student at school? (why?)
4. How happy are you at school? (probe: How embedded do they feel in the school? To what extent to they feel they belong/fit in? What are the best and worst aspects of your school?)
5. Since last March, how much have you been away from school/classroom? (prompt: 1^st^ lockdown, any autumn term 2020 disruptions, 2021 lockdown)
6. How was your experience of learning away from school? (probe: what was offered, how it happened at home, parental support, IT) What did you like and dislike?
   *(If they attended school during lockdowns, ask how was the experience & likes/dislikes)*
7. Do you feel you have learned or done as much learning when you have been away from school? How do you feel about that? (probe: any concerns about falling behind, grades, exams, friendship, future)
8. Do you know if anyone from your school (staff/pupils/parents) got ill from coronavirus?
   *If yes* – How did that make you feel?
9. How much did/do people at school talk about the virus? (probe: teachers, classmates, topics) Any changes inside the school building or classrooms since last spring? (school entrances, desk arrangements, hand washing, masks, etc.)
10. Do you remember what were people’s reactions at school when the virus first started a year ago in Asia? (probe: any misconceptions or racism)
11. Are there issues with racism at your school? (probe: if you have experienced or seen any)

## Friends & spare time

1. What are your friends like at school?
   (probe: ethnicity, background, interests, etc. Are they are similar to you in academic abilities, ethnicities, class?)
2. Do you have other people you regularly see or talk to, say friends or relatives, outside school?
   (prompt: age, ethnicities, family background, interest, what you do with them, etc.)
3. What do you like to do in your spare time? What are you interests/hobbies/out of school activities? Did the lockdown/restrictions affect what you want to do?
4. How did you keep in touch with your friends/relatives during the lockdowns/when you couldn’t see them?

***Family***

1. How are your parents doing? (probe health & general wellbeing)
2. What have your parents been up to since last March? (prompt: travelling to work, working at home, caring for others, home schooling, furloughed, looking for work, etc.) How has the pandemic affected their work/lives?
3. (if applicable) How about other family members (grandparents, siblings, etc.)? How have they been? (probe health & general wellbeing)
4. What languages do you all speak at home? Do your family members speak other languages besides English? (prompt: If yes, do you understand or speak any (x)?)
5. Do you have extended families outside of the UK? How are they doing there?

***Self, London & COVID***

1. How are you doing at the moment? (probe general wellbeing and health) How were you last year? (prompts: when the virus started, during lockdowns)
2. Did you go outside of your home during the lockdowns? (If yes, where? E.g. parks, shops) How did you feel? (e.g. staying home, going outside, exercise)
3. Do you feel comfortable going outside, walking around your home area? Have you gone outside your home area since last March? (If yes, probe to where)
4. Did you go to other parts of London much before the pandemic? How do you feel about going to places like Central London or other parts of London in the near future, e.g. this summer?
5. How do you feel about being someone growing up in London with (X) heritage? (normal, special, easy, hard, etc.? Why?)
6. People often describe London as a very multicultural, diverse city, welcoming to everyone, e.g. during the London Olympics. What do you think? (probe any changes since the pandemic)
7. Would you call yourself a Londoner? Or how would you normally describe yourself? (probe different identities answers)
8. It has been a very difficult year for so many people in London and the UK. Could you just share a few final thoughts on how you feel this is impacting you, your life, aspirations and future? (Prompts: this might be emotionally, socially, physical health, financially, and in terms of feelings about the future)
9. What could anyone – say school, the council, health services, or anywhere, could have done or can do to make things a bit better for you and your family?
10. Just one final question – if you were offered the vaccine for COVID, would you consider taking it? (assure it’s OK if they don’t know)

(Ask if anything they would like to add)
